# The Sub-Pulmonary Left Ventricle in Patients with Systemic Right Ventricle, the Paradoxical Neglected Chamber: A Cardiac Magnetic Resonance Feature Tracking Study

**DOI:** 10.3390/jcm13206033

**Published:** 2024-10-10

**Authors:** Sofia Piana, Alice Pozza, Annachiara Cavaliere, Anna Molinaroli, Irene Cattapan, Jennifer Fumanelli, Martina Avesani, Elena Reffo, Giovanni Di Salvo

**Affiliations:** 1 Paediatric Cardiology Unit, Department of Women and Children’s Health, University Hospital of Padua, 35128 Padua, Italy; sofia.piana@studenti.unipd.it (S.P.); alice.pozza@aopd.veneto.it (A.P.); anna.molinaroli@studenti.unipd.it (A.M.); irene.cattapan@aopd.veneto.it (I.C.); jennifer.fumanelli@aopd.veneto.it (J.F.); martina.avesani@aopd.veneto.it (M.A.); elena.reffo@aopd.veneto.it (E.R.); 2 Pediatric Radiology, Neuroradiology Unit, University Hospital of Padua, 35128 Padua, Italy; annachiara.cavaliere@aopd.veneto.it

**Keywords:** subpulmonary left ventricle, systemic right ventricle, global longitudinal strain, cardiac magnetic resonance, feature tracking

## Abstract

**Background/Objective**: The impact of subpulmonary left ventricle (LV) dysfunction in patients with a systemic right ventricle (SRV) is insufficiently characterized, with only a few studies suggesting its prognostic significance. Additionally, its evaluation through imaging techniques is a challenge. To assess the correlation between quantitative cardiac magnetic resonance-feature tracking (CMR-FT) data and the risk of clinical events related to the natural history of SRV failure. **Methods**: In this cross-sectional study, 21 patients with a diagnosis of transposition of the great arteries (TGA) and atrial switch operation (AtSO) or congenitally corrected transposition (ccTGA) were recruited. All participants underwent CMR-FT analysis. Considered clinical events included NYHA class deterioration (from I-II to III-IV), increased diuretic therapy, arrhythmias, sudden cardiac death, and hospitalizations. **Results**: The cohort consisted of 52.4% males (mean age: 25.4 ± 11.9 years). Eleven patients were diagnosed with ccTGA. Of the 10 patients with TGA post-AtSO, 50% had undergone Mustard repair. Clinical events occurred in 11 patients, with 47.6% experiencing hospitalizations and 28.6% developing arrhythmias. Left ventricular global longitudinal strain (LV GLS) was significantly associated with event-risk in both univariate and multivariate analyses (*p* = 0.011; *p* = 0.025). A cut-off value of LV GLS > −19.24 was proposed to stratify high-risk patients (*p* = 0.001). **Conclusions**: Our study confirms the role of subpulmonary LV function in determining outcomes of SRV patients. The assessment of LV GLS by using CMR-FT could significantly enhance clinical management during follow-up.

## 1. Introduction

Patients with a systemic right ventricle (SRV) and biventricular physiology, like those with congenitally corrected transposition of great arteries (ccTGA) or transposition of the great arteries (TGA) following an atrial switch operation (AtSO), such as a Mustard or Senning operation, represent ≈10% of all congenital heart diseases (CHDs), and their prognosis remains inadequately understood [1,2,3].

While the role of SRV function in the outcome and exercise capacity is well established, the impact of subpulmonary left ventricular (LV) dysfunction is less clear, with only a few studies suggesting a possible role in the outcome of these patients [4,5]. In addition, because of geometrical distortion, reduced afterload and ventricular interactions, the method of evaluating subpulmonic LV function in patients with SRV is not fully established.

Cardiac magnetic resonance (CMR) is considered the gold standard for volume quantification and ejection fraction (EF) for both left and right ventricles in CHDs [6].

Feature tracking (FT) techniques allow for the measuring of myocardial strain in CMR images. Myocardial strain is a geometry independent index that is not affected by tethering from adjacent segments or ventricular interaction and less affected by loading conditions compared with EF [7,8].

The primary objective of this study was to calculate CMR-FT derived strain of both the systemic right ventricle and sub-pulmonary left ventricle in patients with SRV and biventricular physiology. The secondary objective was to assess the correlation between these data and the risk of clinical events related to the natural history of SRV failure.

## 2. Materials and Methods

### 2.1. Study Population

This cross-sectional study was conducted on 21 patients with SRV and biventricular physiology, who were regularly followed at the Pediatric Cardiology Unit of the University Hospital of Padua (Department of Women’s and Children’s Health). They were invited to participate in the study and provided their informed consent. Data collection occurred consecutively between November 2022 and May 2024.

Patients were considered eligible for this study in the presence of SRV and biventricular physiology (TGA post-atrial switch operation and ccTGA). Only patients who underwent a CMR study with feature tracking analysis were included in the study.

Patients with pacemakers (PM), implantable cardioverter defibrillators (ICD), or any other devices incompatible with CMR were excluded from the study. Patients suffering from claustrophobia were excluded. Patients who had never undergone CMR due to compliance issues were also excluded from the study.

Patients’ records were reviewed from the medical platform of Padua University Hospital. Clinical data extracted from medical records included height, weight, body surface area (BSA) according to the Mosteller formula, body mass index (BMI), New York Heart Association (NYHA) class, history of previous clinical events (as will be defined in Section 2.2), and pharmacological treatment, including β-blockers, ACE inhibitors, sartans, diuretics, sacubitril/valsartan, gliflozins, antiarrhythmics, anticoagulants, and antiplatelets.

All data were collected while maintaining confidentiality and were anonymized for statistical analysis. All the information collected was part of routine care. The study was submitted to the attention of the HIT Research Centre Ethical Committee of the University of Padua (Protocol No. 2024_247, 2 May 2024).

### 2.2. Clinical Events

Considered clinical events related to SRV failure were defined as follows: (a) hospitalizations; (b) arrhythmic events (detected either as clinical events requiring hospitalization or during routine Holter ECG monitoring); (c) worsening of NYHA class to III-IV; (d) implementation of diuretic therapy [9,10,11]; (e) sudden cardiac death.

### 2.3. CMR Imaging

Each examination was conducted by a pediatric cardiologist specialized in CMR imaging (ER) and a pediatric radiologist (AC); the investigation was carried out at the Pediatric Neuroradiology Unit of the University Hospital of Padua.

The images were acquired using the same MRI machine (Achieva 1.5T, Philips Healthcare Medical System; Best, The Netherlands).

The standard CMR protocol for evaluating patients with SRV and biventricular physiology includes the following sequences: real-time localization imaging in three axes, non-ECG-gated and free-breathing; cine steady-state free precession (SSFP) sequences, ECG- and respiratory-gated; phase contrast; whole-heart isotropic 3D SSFP imaging; magnetic resonance angiography sequences. Moreover, all patients were administered gadolinium at a dose of 0.2 mmol/kg to detect ventricular wall fibrosis using LGE in both 4-chamber long-axis (4C BTFE) and short-axis views (SA BTFE) [12,13].

**Figure 1:**
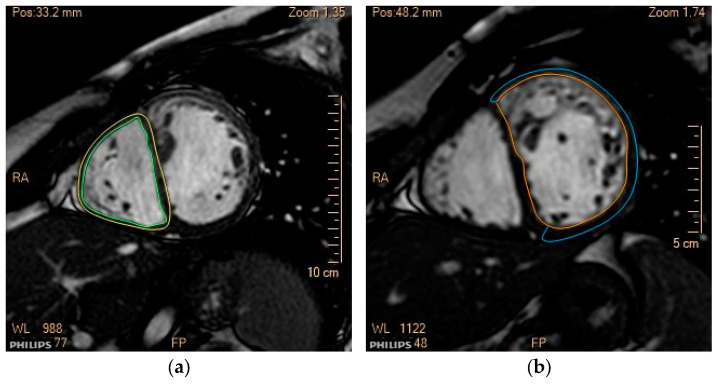
Quantification of the volumes of the sub-pulmonary left ventricle (**a**) and systemic right ventricle (**b**). Calculation of end-diastolic volumes (EDV) and myocardial mass in short axis view (SA BTFE). Trabeculae and papillary muscles were excluded from the calculation. Green line: endocardial border LV; Yellow line: epicardial border LV; Orange line: endocardial border sRV; Blue line: epicardial border sRV.

The analysis of volumes and biventricular ejection fraction was performed using Philips Intellispace Cardiovascular software (Version 7.0). The endocardial and epicardial borders of the SRV and the sub-pulmonary left ventricle (LV) were manually traced in the cineSSFP short-axis sequences, in both the end-diastole and end-systole. Trabeculations and papillary muscles were excluded from the calculation (Figure 1). Measurements were conducted by the same operator (ER) to ensure data consistency.

The following parameters were calculated for both SRV and LV: end-diastolic volume (EDV), end-diastolic volume indexed to BSA (EDVi), end-systolic volume (ESV), end-systolic volume indexed to BSA (ESVi), ejection fraction (EF), stroke volume (calculated as the difference between end-diastolic and end-systolic volume), stroke index (calculated as the difference between indexed end-diastolic and end-systolic volumes), cardiac index ((stroke volume × heart rate/BSA)/1000), and the ratio between SRV and LV EDV.

Additionally, the presence of systemic tricuspid regurgitation (TR) was evaluated in cineSSFP short-axis sequences. The severity of TR was quantified based on the percentage of regurgitant fraction (RF): mild TR for RF < 20%; moderate TR for RF 20–40%; severe TR for RF > 40%.

For both ventricles, myocardial strain was assessed using feature tracking (FT) applied to SSFP cine images during post-processing with dedicated software (Qstrain, Medis Suite Version 4.0.38.4, Leiden, The Netherlands). All calculations were performed by the same operator (SP) to enhance data consistency.

Global longitudinal strains were assessed in 4C BTFE, while global circumferential and radial strains were assessed in SA BTFE.

Images where the walls of both ventricles were clearly delineated were selected to ensure accurate myocardial strain calculation.

The endocardial and epicardial borders were manually traced in end-diastole and then applied to all cardiac cycle phases using an automatic detection algorithm. The accuracy of the borders was checked in all cardiac phases and manually adjusted if necessary. Trabeculations and papillary muscles were excluded.

### 2.4. Statistical Analysis

Normally distributed continuous variables are reported as mean ± standard deviation, while non-normally distributed variables are expressed as the median and interquartile range (IQR). The Shapiro–Wilk test was used to assess the normality of continuous variables. Categorical variables were expressed as percentages.

Initially, a linear regression model was employed. Subsequently, a univariate analysis was conducted. Student’s *t*-test for independent samples was used if normality and homogeneity of variances (tested with Levene’s test) were confirmed, Welch’s test when normality was confirmed but homogeneity of variances was not, and the Mann–Whitney U test when normality was not confirmed. Fisher’s exact test was employed to analyze categorical variables.

Significant variables from the univariate analysis were subjected to a multivariate analysis using the MANCOVA test.

The level of statistical significance for all tests was set at *p* ≤ 0.050.

Data were analyzed using the statistical software Jamovi Version 2.5.5 (2024).

Significant parameters from both the univariate and multivariate MANCOVA tests were used for binary logistic regression. Then, ROC curves were generated to determine the optimal cut-off values. The cut-off point was calculated according to the Youden index to optimize sensitivity (Se) and specificity (Sp); subsequently, the AUC (Area Under the Curve) value and the corresponding real cut-off values were calculated using automated software. This part of the analysis was performed using MedCalc software (MedCalc Software Ltd., version 22.023, Ostend, Belgium).

## 3. Results

### 3.1. Population

According to our selection criteria, we identified 31 patients with SRV and biventricular physiology. Of these, 10 were excluded: five patients because they did not undergo CMR; four patients had complete atrioventricular block and were fitted with non-CMR-compatible pacemakers; one patient was lost to follow-up.

Consequently, the study population comprised 21 patients.

Feature tracking (CMR-FT) was technically feasible in all 21 patients for both the systemic right ventricle and the subpulmonary left ventricle.

### 3.2. Clinical Data

In our cohort, 11 patients were male (52.4%) and 10 female (47.6%).

Ten patients were diagnosed with TGA at birth and underwent AtSO (47.6%, 5 Mustard and 5 Senning), while 11 were affected by ccTGA (52.4%). The median age at which the AtSO was performed was 5.5 months (IQR 4.3–11.3).

At the last visit, the mean age was 25.4 ± 11.9 years. The median age of patients with ccTGA was lower compared to those who underwent the AtSO (19 years (IQR 16.0–30.0) vs. 30 years (IQR 22.5–35.8)). Furthermore, a significant percentage of the patients in the study (*n* = 15, 71.4%) were aged between their second and third–fourth decades of life at the last visit.

Nine patients were found to be overweight (42.9%), defined as having a BMI between 25 and 30 kg/m^2^, while one patient suffered from mild to moderate obesity (4.8%).

Anamnestic and clinical features of the patients are summarized in Table 1.

Thirteen patients (61.9%) were on pharmacological therapy at the last visit. The most frequently used medications were beta-blockers (*n* = 5, 23.8%), ACE inhibitors (*n* = 6, 28.6%) and angiotensin II receptor blockers (*n* = 4, 19.0%). In our cohort, two patients were on diuretic therapy (9.5%). Two patients (9.5%) included in the study were treated with sacubitril/valsartan. Two patients (9.5%) were on antiarrhythmic therapy, both with cordarone, for complex ventricular arrhythmias. Two patients (9.5%) were on anticoagulant therapy and two patients (9.5%) were on antiplatelet therapy.

Pharmacological treatments at the last visit are summarized in Table 2.

### 3.3. Clinical Events

Eleven out of twenty-one patients (52.4%) had clinical events, and seven patients experienced multiple events.

Hospitalizations (*n* = 10, 47.6%) and arrhythmic episodes (*n* = 6, 28.6%) comprised most of the clinical events.

The reasons for hospitalization included arrhythmic episodes (*n* = 7), post-AtSO complications (*n* = 2), and heart failure (*n* = 1). Among the patients with post-AtSO complications, one experienced an occlusion of the pulmonary venous baffle, while the other had a minor baffle leak in association with pulmonary arterial hypertension with a mixed pre- and post-capillary component.

The main arrhythmic events recorded included atrial flutter (*n* = 3) and ventricular arrhythmias (*n* = 3). Additionally, one patient had an episode of paroxysmal supraventricular tachycardia, one patient had atrial fibrillation, and one patient had a complete atrioventricular block.

No patients were classified in NYHA class III or IV. Ten patients were classified as NYHA I (47.6%) and eleven patients as NYHA II (52.4%).

One patient had an episode of sudden cardiac arrest, after which an ICD was implanted.

Three patients adjusted their heart failure therapy by either increasing the dose of diuretics (*n* = 1) or adding HF medications (*n* = 2).

The clinical events are summarized in Table 3.

### 3.4. CMR Imaging Data

All patients (*n* = 21) underwent CMR imaging between May 2019 and November 2023. The mean age of the participants at the time of the examination was 25.1 years ± 11.7. Quantitative CMR imaging data are summarized in Table 4.

Patients with previous clinical events had larger SRV EDV and ESV and lower EF compared to patients without events (SRV EDV 192.0 mL ± 58.5 vs. 173.8 mL ± 61.5, *p* = 0.496; SRV ESV 102.9 mL ± 38.9 vs. 82.4 mL ± 35.4, *p* = 0.223; SRV EF 47.4% ± 7.4 vs. 53.4% ± 6.9, *p* = 0.068). This finding aligns with the natural history of the SRV.

The only significant parameter in the univariate analysis was SRV ESVi (*p* = 0.050). It can be observed that patients who experienced clinical events appear to have a higher SRV ESVi compared to patients without events (58.7 mL/m^2^ ± 15.5 vs. 46.1 mL/m^2^ ± 11.7).

LV EDV and LV ESV values were reduced in patients who had experienced previous clinical events compared to those who had not (LV EDV 119.4 mL ± 47.6 vs. 124.4 ± 47.7, *p* = 0.812; LV ESV 44.0 mL ± 26.3 vs. 50.5 ± 23.6, *p* = 0.559).

Moreover, patients with events had a higher LV EF compared to the group without events (LV EF 65% ± 7.4 vs. 60.6% ± 8.1, *p* = 0.210).

Comparisons of stroke volume, stroke index, and cardiac index for both the systemic right ventricle and the left ventricle among the groups did not yield statistically significant results. Similarly, there were no significant differences in the SRV EDV/LV EDV ratio.

The study did not reveal any correlation between the presence of fibrosis in the systemic right ventricle (*p* = 0.696) and/or left ventricle (*p* = 0.835) and the risk of major events. However, a higher presence of LV fibrosis was observed in both patient groups.

Additionally, no significant correlation between moderate and severe tricuspid regurgitation (RF > 20%) and the risk of major events was demonstrated (*p* = 1.000).

Qualitative CMR imaging data are summarized in Table 5.

### 3.5. Feature Tracking Data

Results of the analysis are reported in Table 6.

Data analysis indicates that the only parameter significantly associated with the risk of events in the univariate analysis is left ventricle global longitudinal strain (LV GLS).

In the studied population (*n* = 21), the mean LV GLS value was −21.1 ± 6.8, and patients who experienced clinical events related to SRV failure had a less negative LV GLS value compared to those without events (−17.7 ± 6.3 vs. −24.9 ± 5.3, *p* = 0.011).

**Figure 2:**
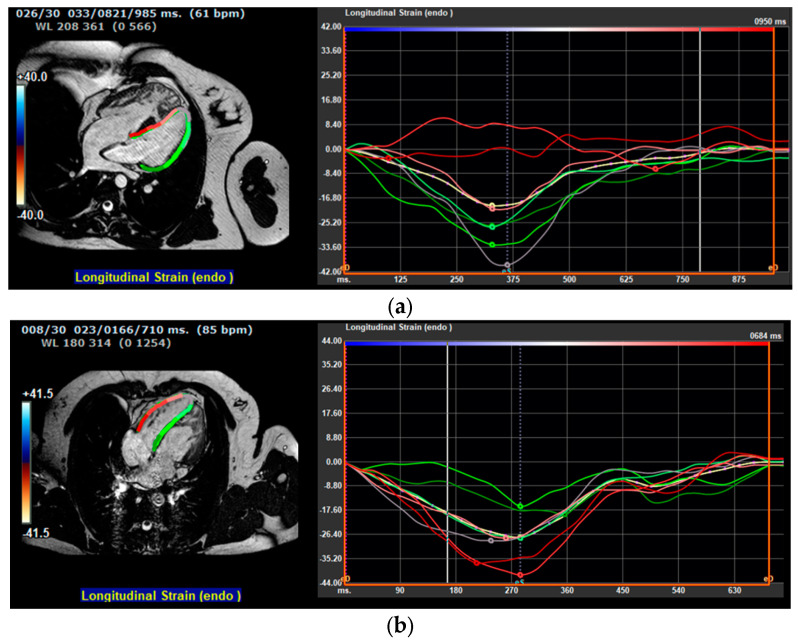
(**a**) LV GLS measurement obtained from a 4C BTFE view is presented for a patient who has undergone AtSO, along with the corresponding strain curve. This patient experienced clinical events: atrial flutter and subsequent hospitalization. (**b**) LV GLS measurement obtained from a 4C BTFE view is presented for a with ccTGA, along with the corresponding strain curve. The patient did not experience clinical events. In both figures (**a**,**b**), the curve representing the mean LV GLS value is depicted in white. The analysis included the average peak strain value of all curves.

In Figure 2a,b, two distinct LV GLS measurements and their associated strain curves are presented.

### 3.6. Multivariate Analysis

SR ESVi and LV GLS were found to be significant in the univariate analysis. These variables were then used to perform the MANCOVA test, which yielded a *p*-value of 0.025 from the multivariate analyses. Given the borderline significance for SRV ESVi, the decision was made to focus the analysis on LV GLS. In Table 7, a comparison of values in the univariate and MANCOVA multivariate analyses is presented.

### 3.7. ROC Curve and Interactive Dot Diagram

**Figure 3:**
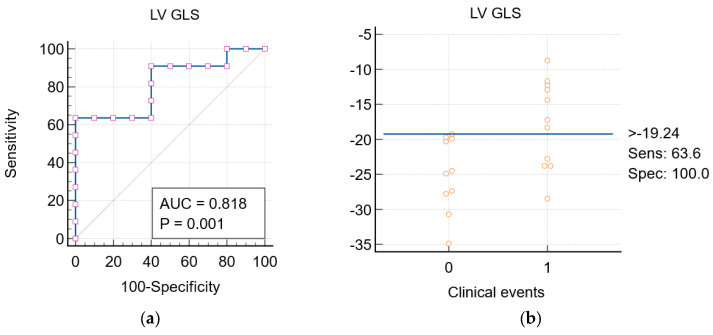
(**a**) LV GLS ROC curve; (**b**) LV GLS interactive dot diagram: the values above the horizontal line are suggested to be associated with a higher risk of clinical events.

For LV GLS, a cut-off value of >−19.24% is proposed. Patients with LV GLS values less negative than this cut-off are at a higher risk for clinical events associated with SRV failure. This threshold demonstrates a Youden’s index of 0.6364, with a sensitivity (Se) of 63.64% (95% CI 30.8–89.1), a specificity (Sp) of 100% (95% CI 69.2–100.0), and an Area Under the Curve (AUC) of 0.818 (*p* = 0.001). These results are reported in Figure 3.

## 4. Discussion

This study is the first to use CMR-FT to analyze the myocardial function of the subpulmonary LV in patients with SRV.

To the best of our knowledge, only a few studies have focused on the subpulmonary LV in patients with SRV and these have primarily used echocardiographic results. In 2021, Surkova et al. (*n* = 157) observed that subpulmonary LV dysfunction detected by echocardiography is associated with increased severity of SRV dysfunction and worsening of the NYHA class [5]. In 2023 (*n* = 180), the same group demonstrated that echocardiographic parameters of the left ventricle (LV-ESDi, LV-FAC) can predict mortality risk and need for transplantation [4]. These studies appear to inaugurate a new perspective on the role of the subpulmonary LV, which has been largely neglected. In detail, these findings raise important questions about the role of the subpulmonary left ventricle and whether its assessment should be included in routine follow-up care.

However, the assessment of subpulmonic LV by using those echo parameters may be limited by geometrical assumption and poor visualization of the morphologically left ventricle. In addition, the used echo parameters are affected by tethering from adjacent segments and ventricular–ventricular interaction. Thus, they may just reflect on the subpulmonic LV and the SRV dysfunction, rather than measure an objective subpulmonic LV dysfunction. Thus, an approach that is not limited by poor visualization, is not affected by geometrical confounder, independent from ventricular interaction, and not affected by loading conditions, may be of value to fully assess the role of the subpulmonic LV. To overcome all these limitations, we used CMR, which is not affected by poor image quality, and we used an FT-derived strain, a parameter that is not affected by geometry, load conditions or ventricular interactions.

Both univariate and multivariate analyses demonstrated a statistical significance of LV GLS (*p* = 0.011; *p* = 0.025) in predicting the risk of clinical events.

Our findings indicate that, even with preserved ejection fraction, a LV GLS > −19.24 could identify latent left ventricle dysfunction. This may be due to the load dependency and influence of geometry in the assessment of EF even by CMR, the gold standard. Santens et al. (2022) have also noted that standard follow-up parameters often fail to detect preclinical dysfunction and deterioration [14]. Our data suggest incorporating the evaluation of the subpulmonary LV in the routine follow-up of these patients, including an evaluation of LV GLS by CMR-FT or by echo in the presence of a good acoustic window.

Our results showed that SRV patients with clinical events had smaller LV EDV and ESV (119.4 ± 47.6 mL vs. 124.4 ± 47.7 mL). This finding is in contrast with the results of Santens et al. In their study (*n* = 33), higher values of LV EDVi and LV ESVi (assessed by CMR both at rest and under stress) were associated with an increased risk of SRV failure, arrhythmias, and death [14]. However, compared with our study, Santens’ group included a larger proportion of patients with Mustard/Senning repairs (58%), older age (37 ± 8 years) and a higher number of cases with moderate/severe tricuspid regurgitation (63%). Thus, the results from Santens et al. reflect a more advanced stage of the disease compared to our study. In this regard, our data suggest that subpulmonary LV dysfunction is associated with events even in an earlier stage of the disease and thus plays an important role in risk stratification.

Moreover, we noted that patients with events had a higher LV EF compared to the group without events (65.0 ± 7.4% vs. 60.6 ± 8.1%). It is known that ejection fraction may not be a sufficiently sensitive parameter to detect changes in ventricular function, being affected by afterload and geometry [15]. For this reason, myocardial strain has been introduced as an intrinsic index of myocardial contractility and function, and our data confirm the higher sensitivity of myocardial strain in detecting early subclinical abnormalities [16,17,18].

### Limitations

The primary limitation of this study is the relatively small sample size of the studied cohort. However, this reflects the prevalence of the disease (10% of all CHDs) and the selection criteria that were applied.

The calculation of myocardial strain using feature tracking software can be relatively time-consuming. However, this process can be significantly expedited with experience.

Furthermore, these results require both intra-operator and inter-operator evaluations to assess data concordance. Therefore, we call for multicenter studies with larger sample sizes to pool expertise and compare results. Such studies will clarify the reproducibility of this parameter and further validate these findings. Additionally, long-term follow-up is necessary to confirm the prognostic significance of this parameter.

## 5. Conclusions

This study has identified an important parameter, the left ventricular global longitudinal strain (LV GLS), which may have significant prognostic value for patients with a systemic right ventricle (SRV). Feature tracking software may overcome some structural limitations often associated with echocardiography in assessing subpulmonary left ventricle function, so its use could be suggested as part of routine follow-ups.

The proposed cut-off value of LV GLS > −19.24 provides a promising reference threshold to identify patients at high risk of SRV failure-related events. Early recognition of LV dysfunction could lead to improved therapeutic strategies, thereby optimizing clinical management and outcomes in this population.

## Figures and Tables

**Table 1:** Anamnestic and clinical features of the population.

Anamnestic and Clinical Data	Sample Size (*n* = 21)
Age at last visit (years)	25.4 ± 11.9
**Gender, *n* (%)**	
Male	11 (52.4%)
Female	10 (47.6%)
**Primary cardiac diagnosis**	
ccTGA, *n* (%)	11 (52.4%)
TGA, *n* (%)	10 (47.6%)
s/p Mustard	5 (23.8%)
s/p Senning	5 (23.8%)
Age at operation (months)	5.5 (IQR 4.3–11.3)
**Associated cardiac abnormalities**	
ASD at birth, *n* (%)	2 (9.5%)
VSD at birth, *n* (%)	9 (42.9%)
Pulmonary stenosis at birth, *n* (%)	7 (33.3%)
Pulmonary atresia at birth, *n* (%)	3 (14.3%)
DORV, *n* (%)	1 (4.8%)
Ebstein’s anomaly, *n* (%)	2 (9.5%)
Straddling tricuspid valve, *n* (%)	1 (4.8%)
**Cardiac situs, *n* (%)**	
Levocardia	17 (81.0%)
Dextrocardia	4 (19.0%)
**Anthropometric measurements**	
Height (m)	1.7 (IQR 0.9–1.8)
Weight (kg)	66.6 ± 21.7
BSA (m^2^)	1.8 (IQR 0.6–2.3)
BMI (kg/m^2^)	24.4 ± 0.4

**Table 2:** Pharmacological therapies administered at the last visit.

Pharmacological Therapies	Sample Size (*n* = 21)
Ongoing therapy, *n* (%)	13 (61.9%)
Beta-blockers	5 (23.8%)
ACE-inhibitors	6 (28.6%)
Sartans	4 (19.0%)
Diuretics	2 (9.5%)
Sacubitril/valsartan	2 (9.5%)
SGLT2 inhibitors	0 (0%)
Antiarrhythmics	2 (9.5%)
Anticoagulants	2 (9.5%)
Antiplatelets	2 (9.5%)

**Table 3:** Clinical events related to SRV failure in our cohort.

Clinical Events	Sample Size (*n* = 21)
Events, *n* (%)	11 (52.4%)
Hospitalizations	10 (47.6%)
Arrhythmias	6 (28.6%)
Sudden cardiac death	1 (4.8%)
Implementation of anti-failure therapy	3 (14.3%)
NYHA III-IV	0 (0%)

**Table 4:** Quantitative CMR imaging data: mean values and univariate analysis. The first column reports the mean values of the following parameters in the study population. The second and third columns display the mean values for the subpopulations with and without events, respectively. The fourth column presents the *p*-value associated with the univariate analysis.

	All (*n* = 21)	Patients with Previous Events (*n* = 11)	Patients without Previous Events (*n* = 10)	*p*-Value * (*p* ≤ 0.050)
SRV EDV (mL)	183.0 ± 59.1	192.0 ± 58.5	173.8 ± 61.5	0.496
SRV EDVi (mL/m^2^)	105.0 ± 20.3	110.7 ± 20.1	98.4 ± 19.4	0.169
SRV ESV (mL)	93.2 ± 37.8	102.9 ± 38.9	82.4 ± 35.4	0.223
SRV ESVi (mL/m^2^)	52.7 ± 14.9	58.7 ± 15.5	46.1 ± 11.7	**0.050**
SRV EF (%)	50.2 ± 7.61	47.4 ± 7.4	53.4 ± 6.9	0.068
SRV stroke index (mL/m^2^)	52.1 ± 11.0	52.0 ± 9.4	52.3 ± 13.0	0.952
SRV stroke volume (mL)	90.2 ± 27.1	89.0 ± 24.1	91.4 ± 31.5	0.847
SRV cardiac index (L/min/m^2^)	3.7 ± 0.9	3.0 ± 0.6	4.0 ± 1.0	0.077
LV EDV (mL)	122.0 ± 46.5	119.4 ± 47.6	124.4 ± 47.7	0.812
LV EDVi (mL/m^2^)	69.3 ± 20.9	68.3 ± 25.0	70.4 ± 16.4	0.822
LV ESV (mL)	47.1 ± 24.7	44.0 ± 26.3	50.5 ± 23.6	0.559
LV ESVi (mL/m^2^)	26.5 ± 12.3	26.8 ± 14.3	29.6 ± 8.7	0.587
LV EF (%)	62.9 ± 7.9	65.0 ± 7.4	60.6 ± 8.1	0.210
LV stroke index (mL/m^2^)	42.8 ± 10.7	45.7 ± 8.5	43.3 ± 10.6	0.853
LV stroke volume (mL)	74.7 ± 25.3	75.4 ± 23.6	73.9 ± 28.4	0.896
LV cardiac index (L/min/m^2^)	3.0 ± 0.9	2.8 ± 0.8	3.3 ± 1.0	0.207
SRV EDV/LV EDV	1.5 (IQR 0.8–3.4)	1.6 (IQR 1.5–1.9)	1.4 (IQR 1.2–1.6)	0.173

* Bold *p*-value indicates statistically significant.

**Table 5:** Qualitative CMR imaging data: absolute frequencies and univariate analysis. The first column reports the absolute frequency and the relative percentage of the following qualitative data in the study population. The second and third columns display the absolute frequencies and relative percentages in the subpopulations with and without events, respectively. The fourth column presents the *p*-value associated with the univariate analysis.

	All (*n* = 21)	Patients with Previous Events (*n* = 11)	Patients without Previous Events (*n* = 10)	*p*-Value (*p* ≤ 0.050)
SRV fibrosis, *n* (%)	5 (23.8%)	3 (27.3%)	2 (10.0%)	0.696
LV fibrosis, *n* (%)	10 (47.6%)	5 (45.5%)	5 (50.0%)	0.835
TR, *n* (%)	13 (61.9%)	8 (72.7%)	5 (50.0%)	0.284
Mild	7 (33.3%)	5 (45.5%)	2 (20.0%)	
Moderate	4 (19.0%)	3 (27.3%)	1 (10.0%)	
Severe	2 (9.5%)	0 (0%)	2 (20.0%)	
Moderate-severe TR, *n* (%)	6 (28.6%)	3 (27.3%)	3 (27.3%)	1.000

**Table 6:** Feature tracking data. Mean values in the study population and in the subpopulations with and without events, respectively. Univariate analysis and *p*-values.

	All (*n* = 21)	Patients with Previous Events (*n* = 11)	Patients without Previous Events (*n* = 10)	*p*-Value * (*p* ≤ 0.050)
SRV GLS (%)	−20.2 ± 5.3	−18.6 ± 5.6	−22.0 ± 4.5	0.145
SRV GCS (%)	−22.0 ± 4.3	−21.6 ± 5.3	−22.4 ± 3.1	0.655
SRV GRS (%)	80.2 (IQR 60.1–134.0)	69.0 (IQR 58.7–116.0)	109.1 (IQR 69.2–138.4)	0.605
LV GLS (%)	−21.1 ± 6.8	−17.7 ± 6.3	−24.9 ± 5.3	**0.011**
LV GCS (%)	−24.8 ± 5.4	−26.5 ± 6.2	−23.1 ± 3.9	0.156
LV GRS (%)	60.1 ± 27.6	51.2 ± 20.3	69.9 ± 32.1	0.137

* Bold *p*-value indicates statistically significant.

**Table 7:** Comparison of values in univariate and MANCOVA multivariate analyses. The results are statistically significant, with a borderline significance noted for SRV ESVi (*p* = 0.050).

**Univariate Analysis**	**Dependent Variables**	***p*-Value**
Events	SRV ESVi	0.050
	LV GLS	0.011
**Multivariate Analysis**	**Dependent Variables**	***p*-Value**
Events	SRV ESVi, LV GLS	0.025

## Data Availability

Data sharing is not applicable to this article.

## Abbreviations

CHDs	congenital heart diseases
SRV	systemic right ventricle
LV	left ventricle
ccTGA	congenitally corrected transposition of the great arteries
TGA	transposition of the great arteries
AtSO	atrial switch operation
ASD	atrial septal defect
VSD	ventricular septal defect
DORV	double outlet right ventricle
CMR	cardiac magnetic resonance
CMR-FT	cardiac magnetic resonance-feature tracking
LGE	late gadolinium enhancement
SRV EDV	systemic right ventricle end-diastolic volume
SRV EDVi	systemic right ventricle end-diastolic volume indexed to BSA
SRV ESV	systemic right ventricle end-systolic volume
SRV ESVi	systemic right ventricle end-systolic volume indexed to BSA
SRV EF	systemic right ventricle ejection fraction
LV EDV	left ventricle end-diastolic volume
LV EDVi	left ventricle end-diastolic volume indexed to BSA
LV ESV	left ventricle end-systolic volume
LV ESVi	left ventricle end-systolic volume indexed to BSA
LV EF	left ventricle ejection fraction
TR	tricuspid regurgitation
SRV GLS	systemic right ventricle global longitudinal strain
SRV GCS	systemic right ventricle global circumferential strain
SRV GRS	systemic right ventricle global radial strain
LV GLS	left ventricle global longitudinal strain
LV GCS	left ventricle global circumferential strain
LV GRS	left ventricle global radial strain

## References

[1] Samánek M., Slavík Z., Zborilová B., Hrobonová V., Vorísková M., Skovránek J. (1989). Prevalence, treatment, and outcome of heart disease in live-born children: A prospective analysis of 91,823 live-born children. Pediatr. Cardiol..

[2] Spadotto V., Frescura C., Ho S.Y., Thiene G. (2017). The concept of double inlet-double outlet right ventricle: A distinct congenital heart disease. Cardiovasc. Pathol..

[3] Nartowicz S.A., Jakielska E., Ratajczak P., Lesiak M., Trojnarska O. (2024). Clinical Factors Affecting Survival in Patients with Congenitally Corrected Transposition of the Great Arteries: A Systematic Review and Meta-Analysis. J. Clin. Med..

[4] Surkova E., Constantine A., Xu Z., Segura de la Cal T., Bispo D., West C., Senior R., Dimopoulos K., Li W. (2023). Prognostic significance of subpulmonary left ventricular size and function in patients with a systemic right ventricle. Eur. Heart J.-Cardiovasc. Imaging.

[5] Surkova E., Segura T., Dimopoulos K., Bispo D., Flick C., West C., Babu-Narayan S.V., Senior R., Gatzoulis M.A., Li W. (2021). Systolic dysfunction of the subpulmonary left ventricle is associated with the severity of heart failure in patients with a systemic right ventricle. Int. J. Cardiol..

[6] Di Salvo G., Miller O., Babu Narayan S., Li W., Budts W., Valsangiacomo Buechel E.R., Frigiola A., Bosch A.E.V.D., Bonello B., Mertens L. (2018). Imaging the adult with congenital heart disease: A multimodality imaging approach—Position paper from the EACVI. Eur. Heart J.-Cardiovasc. Imaging.

[7] Sutherland G.R., Di Salvo G., Claus P., D’hooge J., Bijnens B. (2004). Strain and strain rate imaging: A new clinical approach to quantifying regional myocardial function. J. Am. Soc. Echocardiogr..

[8] Stokke T.M., Hasselberg N.E., Smedsrud M.K., Sarvari S.I., Haugaa K.H., Smiseth O.A., Edvardsen T., Remme E.W. (2017). Geometry as a Confounder When Assessing Ventricular Systolic Function: Comparison Between Ejection Fraction and Strain. J. Am. Coll. Cardiol..

[9] Chaix M.A., Dore A., Mondésert B., Mongeon F.P., Roy V., Desrosiers-Gagnon C., Guertin M.-C., White M., Ibrahim R., O’meara E. (2024). Angiotensin receptor-neprilysin inhibitor vs. placebo in congenital systemic right ventricular heart failure: The PARACYS-RV trial. Eur. Heart J..

[10] Fusco F., Scognamiglio G., Merola A., Iannuzzi A., Palma M., Grimaldi N., Sarubbi B. (2023). Safety and Efficacy of Sacubitril/Valsartan in Patients with a Failing Systemic Right Ventricle: A Prospective Single-Center Study. Circ. Heart Fail..

[11] Nederend M., Kiès P., Regeer M.V., Vliegen H.W., Mertens B.J., Robbers-Visser D., Bouma B.J., Tops L.F., Schalij M.J., Jongbloed M.R.M. (2023). Tolerability and beneficial effects of sacubitril/valsartan on systemic right ventricular failure. Heart.

[12] Fogel M.A., Anwar S., Broberg C., Browne L., Chung T., Johnson T., Muthurangu V., Taylor M., Valsangiacomo-Buechel E., Wilhelm C. (2022). Society for Cardiovascular Magnetic Resonance/European Society of Cardiovascular Imaging/American Society of Echocardiography/Society for Pediatric Radiology/North American Society for Cardiovascular Imaging Guidelines for the Use of Cardiac Magnetic Resonance in Pediatric Congenital and Acquired Heart Disease: Endorsed by The American Heart Association. Circ. Cardiovasc. Imaging.

[13] Canan A., Ashwath R., Agarwal P.P., François C., Rajiah P. (2021). Multimodality Imaging of Transposition of the Great Arteries. RadioGraphics.

[14] Santens B., Helsen F., Van De Bruaene A., De Meester P., Budts A.L., Troost E., Moons P., Claus P., Rega F., Bogaert J. (2022). Adverse functional remodelling of the subpulmonary left ventricle in patients with a systemic right ventricle is associated with clinical outcome. Eur. Heart J.-Cardiovasc. Imaging.

[15] Taylor R.J., Moody W.E., Umar F., Edwards N.C., Taylor T.J., Stegemann B., Townend J.N., Hor K.N., Steeds R.P., Mazur W. (2015). Myocardial strain measurement with feature-tracking cardiovascular magnetic resonance: Normal values. Eur. Heart J.-Cardiovasc. Imaging.

[16] Rajiah P.S., Kalisz K., Broncano J., Goerne H., Collins J.D., François C.J., Ibrahim E.-S., Agarwal P.P. (2022). Myocardial Strain Evaluation with Cardiovascular MRI: Physics, Principles, and Clinical Applications. RadioGraphics.

[17] Scatteia A., Baritussio A., Bucciarelli-Ducci C. (2017). Strain imaging using cardiac magnetic resonance. Heart Fail. Rev..

[18] Sperlongano S., D’Andrea A., Mele D., Russo V., Pergola V., Carbone A., Ilardi F., Di Maio M., Bottino R., Giallauria F. (2021). Left Ventricular Deformation and Vortex Analysis in Heart Failure: From Ultrasound Technique to Current Clinical Application. Diagnostics.

